# Mechanic’s hands in a woman with undifferentiated connective tissue disease and interstitial lung disease – anti-PL7 positive antisynthetase syndrome: a case report

**DOI:** 10.1186/s13256-015-0571-2

**Published:** 2015-04-15

**Authors:** Ellen De Langhe, Jan Lenaerts, Xavier Bossuyt, Rene Westhovens, Wim A Wuyts

**Affiliations:** 1 Department of Rheumatology, University Hospitals Leuven, Herestraat 49, Leuven, 3000 Belgium; 2 Department of Laboratory Medicine, University Hospitals Leuven, Herestraat 49, Leuven, 3000 Belgium; 3 Department of Pulmonary Medicine, University Hospitals Leuven, Herestraat 49, Leuven, 3000 Belgium

**Keywords:** Antisynthetase syndrome, Connective tissue diseases, Dermatomyositis, Pulmonary fibrosis

## Abstract

**Introduction:**

Interstitial lung disease can be idiopathic or occur in the setting of connective tissue diseases. In the latter case it requires a different treatment approach with a better prognosis. Interstitial lung disease can precede the onset of typical connective tissue disease features by many years, and therefore meticulous multidisciplinary follow-up is crucial. This case highlights the diagnostic challenge and the need for intensified attention for subtle clinical features when faced with interstitial lung disease in patients with characteristics of a hitherto undifferentiated connective tissue disease.

**Case presentation:**

A 44-year-old Caucasian woman presented to our pulmonology department with dyspnea, Raynaud’s phenomenon and subtle swelling of fingers and eyelids. Laboratory analysis and autoantibody screening was negative. She was diagnosed with nonspecific interstitial pneumonia with a concurring undifferentiated connective tissue disease. After four years of stable disease, she presented with rapid pulmonary deterioration, myalgia, periorbital edema, arthritis and a cracked appearance of the radial sides of the fingers of both her hands. This clinical sign was recognized as mechanic’s hands and a specific search for the presence of antisynthetase antibodies was performed. She was found to harbor anti-threonyl-tRNA synthetase antibodies. A diagnosis of antisynthetase syndrome was made and she was treated with glucocorticoids and immunosuppressives.

**Conclusions:**

This case highlights the difficulty in fine-tuning the diagnosis when confronted with a patient with interstitial lung disease and the suspicion of an underlying, yet undifferentiated connective tissue disease. There is a strong need for clinical multidisciplinary follow-up of these patients, with a high level of alertness to rare and specific clinical signs. The diagnosis of the underlying connective tissue disease profoundly influences the management of the interstitial lung disease. Recent data stress that identification of the autoantibody specificity allows for further prognostic stratification and therefore should be pursued.

## Introduction

Interstitial lung disease (ILD) can occur in the setting of almost all connective tissue diseases (CTDs). CTD-associated ILD warrants specific treatment strategies, based on glucocorticoids and immunosuppressive agents and confers a better prognosis [1]. The recognition of the underlying CTD is therefore crucial but is often hampered and the diagnosis is missed. This can be attributed to various reasons. For one, it is known that ILD may precede the appearance of typical extrathoracic CTD features by many years [2]. Furthermore, the initial presentation of the CTD may be subtle and easily overlooked when no meticulous long-term multidisciplinary follow-up is organized. Moreover, many autoantibodies are rare and not routinely tested, and a negative screening assay for antinuclear antibodies (ANA) and anti-histidyl-tRNA synthetase (anti-Jo-1) antibodies may mislead the treating clinician. We present a case of a woman, diagnosed with nonspecific interstitial pneumonia (NSIP) with the concurrent suspicion of an underlying CTD in 2008, but not meeting classification criteria for any specific CTD. After four years of stable disease she presented with sudden pulmonary and systemic deterioration with the appearance of mechanic’s hands, pointing to a diagnosis of underlying antisynthetase syndrome (ASS) and resulting in reorientation of therapeutic strategies.

## Case presentation

In 2008, a 44-year-old Caucasian woman presented to our pulmonary out-patient clinic with progressive dyspnea and cough. She mentioned recent swelling of her fingers and eyelids. Furthermore, Raynaud’s phenomenon was present for many years and she complained of a pruritic rash on the inner side of her right thigh and widespread arthralgia.

**Figure 1:**
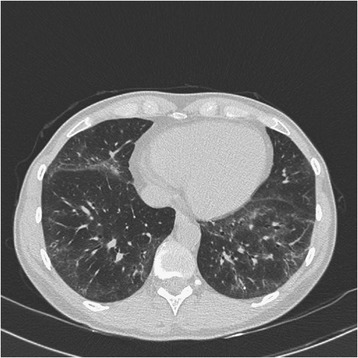
High-resolution computed tomography image. Image compatible with nonspecific interstitial pneumonia showing ground-glass opacities in both lung bases.

On clinical examination we found bibasilar crackles, puffy fingers and swollen eyelids. On her right thigh we observed a discrete macular rash. Pulmonary function tests noted normal lung volumes and a reduced diffusion capacity: forced vital capacity (FVC) 85%; forced expiratory volume in 1 second (FEV1) 85%; carbon monoxide diffusion capacity (DLCO) 46%. High-resolution computed tomography (HRCT) demonstrated ground-glass opacities in both lung bases, with limited honeycombing, compatible with NSIP (Figure 1). Bronchoalveolar lavage (BAL) analysis revealed increased cellularity (37.04×10^6^ cells/microliter) with increased lymphocyte (25.6%) and eosinophil count (12.4%). Laboratory analysis noted mild inflammation (C-reactive protein, CRP, 12mg/L), normal liver tests and a normal creatine kinase level. ANA were negative and no anticytoplasmic staining was detected on indirect immunofluorescence (IIF). Rheumatoid factor and anti-cyclic citrullinated protein antibodies were negative. No specific search for the presence of antisynthetase antibodies was performed. Capillaroscopy showed only aspecific findings with two megacapillaries, insufficient for a diagnosis of systemic sclerosis.

She was diagnosed with NSIP with a high suspicion of an underlying CTD, because of the presence of Raynaud’s phenomenon, puffy fingers, a NSIP pattern on HRCT and the lymphocytic BAL formula. However, the absence of ANA and the absence of a clear signature on capillaroscopy led to the diagnosis of undifferentiated CTD (UCTD).

**Figure 2:**
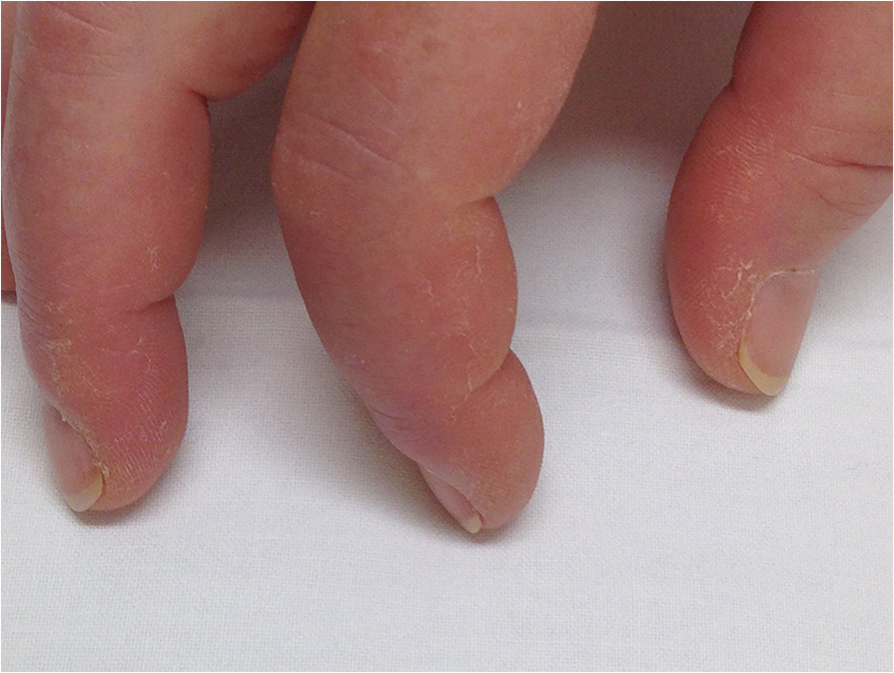
Mechanic’s hands. Cracking and hyperkeratosis of the radial surface of digits one to three.

She was monitored with regular clinical, laboratory, radiological and lung function evaluations. All parameters remained stable for four years. Suddenly, she presented with progressive dyspnea, fever, night sweats, weight loss, myalgia and muscle weakness, arthralgia, swollen eyelids and cracked fingers (Figure 2). A clinical examination revealed a febrile, discrete hypotensive and tachycardic patient (39°C, blood pressure 101/57mmHg, pulse rate 101 beats per minute). We noted a heliotropic rash, polyarthritis of hand proximal interphalangeal joints as well as proximal and distal muscular weakness and tenderness. The skin on the radial side of her first to third fingers of both hands appeared hyperkeratotic and cracked, compatible with mechanic’s hands. Laboratory analysis documented severe inflammation (erythrocyte sedimentation rate 112mm/hour, CRP 192.2mg/L) and elevated creatine kinase levels (713U/L, normal <=145U/L). IIF showed a negative ANA and a positive anticytoplasmic signal (titer 1/640). Her blood and urine cultures were negative. A chest radiograph showed no evidence for an intercurrent infection. Electromyography studies were compatible with severe myopathy in both proximal and distal muscle groups. Pulmonary function tests revealed severe restriction and severely decreased diffusion capacity (FVC 44%; FEV1 40%; DLCO 31%). A transthoracic echocardiogram showed normal right and left ventricular function with an estimated ejection fraction of 60%. A small pericardial effusion was present (maximal end-diastolic diameter 5mm) without hemodynamic effect. Systolic arteria pulmonalis pressure estimate was 45mmHg. Cardiac magnetic resonance imaging demonstrated discrete contrast enhancement in the basal septum, compatible with previous myocarditis.

This clinical picture was compatible with an ASS and urged the search for antisynthetase antibodies. Anti-Jo-1 antibodies were negative, but more specific testing (immunodot technique, Alphadia, Belgium) revealed the presence of anti-threonyl-tRNA synthetase (anti-PL7) antibodies. She was treated with high-dose glucocorticoids (1mg/kg methylprednisolone with a tapering scheme) in combination with mycophenolate-mofetil. This resulted in normalization of her creatine kinase levels and stabilization of pulmonary function tests.

## Discussion

ASS is a clinical entity characterized by ILD, inflammatory myopathy, arthritis, Raynaud’s phenomenon, fever, mechanic’s hands and the presence of antisynthetase antibodies. The clinical feature of mechanic’s hands is characterized by erythematous and fissured hyperkeratosis on the palmar or lateral aspect of the fingers. It is considered a highly specific clinical sign, present in 28% of patients carrying antisynthetase antibodies [3]. Other features include cardiac involvement, with myocarditis and a reported prevalence of pericarditis of 20 to 50% [4,5] and gastrointestinal involvement (esophageal dysfunction). Antisynthetase antibodies are aminoacyl-transfer RNA synthetase (ARS) antibodies, directed against the enzymes that attach dedicated amino acids to their cognate transfer ribonucleic acid (tRNA in the process of polypeptide synthesis. Theoretically, ARS could be targeting synthetases for all 20 existing amino acids. However, to date, only eight different ARS have been identified, of which anti-Jo-1 is the most prevalent, comprising two-thirds of identified ARS antibodies [6]. Others are less frequent and include anti-alanyl-tRNA synthetase (anti-PL12), anti-PL7, anti-isoleucyl-tRNA synthetase, anti-glycyl-tRNA synthetase, anti-asparaginyl-tRNA synthetase, anti-phenylalanyl-tRNA synthetase and anti-tyrosyl-tRNA synthetase [7]. Detection of these antibodies is crucial. Routine ANA testing is based on IIF and will often remain negative in patients with ASS. ARS antibodies are directed against cytoplasmic antigens and their presence is often suggested, albeit nonspecific, by an anticytoplasmic signal on IIF. The detection of anti-Jo-1 antibodies is performed by a commercially available immunoassay. However, the identification of other ARS antibodies classically requires protein and RNA immunoprecipitation techniques that are not routinely performed in all laboratories and often need a specific inquiry from the treating clinician [7]. Currently, commercial immunodot techniques are available for detection of some ARS, such as anti-PL12 and anti-PL7 antibodies.

The high prevalence of ILD in patients carrying ARS and the need for specific immunosuppressive treatment emphasizes the need for timely detection of this clinical syndrome. This is however often severely hampered by the variable time relation between the onset of ILD and the onset of myositis or other ASS-specific features. ILD can precede (10 to 30%), concur (53 to 70%) or follow (6 to 20%) the onset of myositis [4,5]. Organized multidisciplinary follow-up by both pulmonologists and rheumatologists increases the alertness for subtle clinical signs suggesting an underlying ASS.

When faced with a patient with an ASS, the identification of the underlying ARS antibody specificity is relevant. Increasing recent evidence supports clinical heterogeneity between anti-Jo-1 and non-Jo-1 positive patients with differences in presenting symptoms and prognosis. On the one hand, anti-Jo-1 positive patients more frequently present with muscle and joint involvement with more severe and treatment-resistant myositis. Non-Jo-1 positive patients on the other hand often present with non-myositis CTD symptoms (Raynaud, cutaneous changes, constitutional symptoms) and pulmonary manifestations [3-5,8]. Anti-PL7 and anti-PL12 positive patients cluster together with a 98% prevalence of ILD and even isolated ILD in 62%. The behavior of anti-PL7 and anti-PL12 positive patients is substantially different from anti-Jo-1 positive patients (ILD in 78%) [9], a finding that is shared in other reports [10,11]. Identification of the ARS antibody allows for prognostic stratification. Recent reports indicate that non-Jo-1 positive patients have decreased survival compared to anti-Jo-1 positive patients (5- and 10-year unadjusted survival of 90% and 70% in anti-Jo-1 positive patients, and 75% and 47% in non-Jo-1 positive patients) [8]. In conclusion, anti-PL7 positive patients have a prevalence of ILD of 90 to 100%, with a high prevalence of isolated ILD at presentation, lower frequency of myositis with a better response to therapy, and an overall poorer prognosis compared to anti-Jo-1 positive patients.

We feel that this case is an excellent example to highlight the relevance of an underlying UCTD in a patient presenting with ILD. The presence of CTD features (sicca syndrome, Raynaud’s phenomenon, cutaneous or musculoskeletal symptoms) in a patient with ILD should urge the clinician for further research, especially in the presence of a radiological or histopathological NSIP pattern, female gender or younger age. The diagnosis of UCTD can be made in patients presenting with at least one CTD feature and the presence of autoantibodies or systemic inflammation, but not fulfilling the preset classification criteria for a definite CTD. ILD in a patient with UCTD can be considered “lung-dominant CTD”, a concept that was introduced by Fischer *et al*. [12] and defined as ILD with: (1) a histopathological or radiological pattern of NSIP, usual interstitial pneumonia, lymphocytic interstitial pneumonia, organizing pneumonia or diffuse alveolar damage; (2) insufficient extrathoracic features for a definite CTD diagnosis; (3) no other available etiology for the interstitial pneumonia; and (4) the presence of any of a listed set of autoantibodies or two out of four histopathological features on lung biopsy (lymphoid aggregates with germinal centers, extensive pleuritis, prominent plasmocytic infiltration and dense perivascular collagen). Specific interest has also gone to patients with ILD and the presence of autoantibodies without any CTD feature. Patients with usual interstitial pneumonia (UIP) and the presence of autoantibodies have more ground-glass opacities, more extensive ILD and more honeycombing, but identical survival compared to patients with autoantibody-negative UIP [13]. When compared to healthy controls, patients with autoantibody-positive idiopathic pulmonary fibrosis have identical autoantibody profiles, suggesting that these are antibodies of unknown significance in the ageing patient, without a clear clinical impact [14].

In general, clinicians should be alert for an underlying CTD in patients presenting with ILD. The diagnosis of UCTD or lung-dominant CTD should be suspected in a patient with ILD and the presence of one CTD feature and the presence of autoantibodies, as UCTD can evolve into full-blown CTD during the course of follow-up.

## Conclusions

With this case we want to highlight the fact that the first pulmonary presentation may precede the full-blown CTD picture by many years. This can hamper and delay a final diagnosis and treatment. There is a crucial need for meticulous collaborative follow-up by pulmonologists and rheumatologists in patients with suspected “lung-dominant CTD”. Furthermore this case emphasizes the need to consider ASS in patients with ILD and UCTD, and to screen for ARS antibodies, particularly in the presence of typical clinical features (mechanic’s hands), a negative ANA and a positive anticytoplasmic signal.

## Consent

Written informed consent was obtained from the patient for publication of this case report and accompanying images. A copy of the written consent is available for review by the Editor-in-Chief of this journal.

## Abbreviations

ANA: Antinuclear antibodies
Anti-Jo-1: Anti-histidyl-tRNA synthetase
Anti-PL7: Anti-threonyl-tRNA synthetase
Anti-PL12: Anti-alanyl-tRNA synthetase
ARS: Aminoacyl-transfer RNA synthetase
ASS: Antisynthetase syndrome
BAL: Bronchoalveolar lavage
CRP: C-reactive protein
CTD: Connective tissue disease
DLCO: Carbon monoxide diffusion capacity
FEV1: Forced expiratory volume in 1 second
FVC: Forced vital capacity
HRCT: High-resolution computed tomography
IIF: Indirect immunofluorescence
ILD: Interstitial lung disease
NSIP: Nonspecific interstitial pneumonia
tRNA: Transfer ribonucleic acid
UCTD: Undifferentiated connective tissue disease
UIP: Usual interstitial pneumonia
